# Analysis of Phenomenon of Plasticity Loss of Steel Core Made by Selective Laser Melting Method in Zone of Pressure Mould Conformal Cooling Channel

**DOI:** 10.3390/ma16124205

**Published:** 2023-06-06

**Authors:** Jarosław Piekło, Aldona Garbacz-Klempka

**Affiliations:** Faculty of Foundry Engineering, AGH University of Krakow, Al. Mickiewicza 30, 30-059 Krakow, Poland; jarekp60@agh.edu.pl

**Keywords:** die casting, conformal cooling, strain analysis, microstructural analysis, triaxiality factor, failure criterion, fractography analysis

## Abstract

This paper presents the results of testing the mechanical properties of maraging steel 1.2709 that were obtained by the SLM method under uniaxial and triaxial states of stress. The triaxial state of stress was realised by making circumferential notches in the samples with different radii of rounding. The specimens were subjected to two types of heat treatment, which consisted of ageing at 490 °C and 540 °C for 8 h. The results of the tests that were conducted on the samples were considered as references and compared with the results of the strength tests that were conducted directly on the SLM-made core model. Differences were found between the results of these tests. Based on the experimental results, the relationship between the equivalent strain of the specimen in the bottom of notch ε_eq_ and triaxiality factor η was determined. Function ε_eq_ = f(η) was proposed as a criterion for the decrease in the plasticity of the material in the area of the pressure mould cooling channel. Using the FEM method, equivalent strain field ε_eq_ and triaxiality factor η were determined in the conformal channel cooled core model. Based on the proposed criterion of plasticity loss and the results of numerical calculations, it was shown that the values of equivalent strain ε_eq_ and triaxiality factor η in the core that was aged at 490 °C did not meet this criterion. On the other hand, the values of strain ε_eq_ and triaxiality factor η did not exceed the safety limit when ageing was carried out at 540 °C. The plasticity loss method presented in this paper assumes that the value of the triaxiality factor in the vicinity of the channel is influenced by the shape, cross-sectional dimensions and trajectory of the channel axis. Using the methodology proposed in this paper, it is possible to determine the value of allowable deformations in the cooling channel zone and to determine whether the heat treatment applied to the SLM steel does not cause an excessive reduction in the plastic properties.

## 1. Introduction

Significant advances in the development of 3D printing methods—including SLM (selective laser melting) technology [1]—create the possibility of its application for fabricating tooling parts of pressure casting moulds. The use of the SLM method for fabricating pressure mould parts significantly expands the range of available design solutions for the designed mould cooling system as compared to machining methods. By matching the trajectory of the channels to the shape of the surface of the mould cavity or core, the rate of the heat removal from the solidifying metal is increased, resulting in a more homogeneous temperature field in the casting [2]; also, shrinkage porosity can be largely eliminated, and a fine-grained structure of the casting can be achieved [3,4,5]. This results in a casting with greater mechanical strength and tightness. The use of conformal channels also reduces the cycle time of the pressure machine. Compared to the classical way of designing cooling channels, conformal channels are located closer to the metal¬–mould contact surface and have smaller diameters. The design distances between the surfaces of adjacent channels are also smaller, and the channel trajectory undergoes multiple directional changes [6,7,8]. The above-mentioned features of conformal channels create various types of fabrication, technological, service, and strength problems. Cooling channels with an excessively large diameter (greater than 8 mm) may experience deformation of the top surface during the SLM process. On the other hand, a small channel diameter can cause a layer of contamination to be deposited on the channel surfaces during operation, which narrows the channel clearance. The frequent changes in channel trajectory and the small cross-sectional area characteristic of a conformal cooling system result in a localised increase in stresses caused by the notch effect. Despite the above-mentioned difficulties in making fully usable parts using the SLM method, there has been an increase in the number of applications of this technology in the manufacture of parts that constitute mould cooling systems—especially injection moulds that are intended for the production of plastic components [9]. There is also an increasing number of cases where the SLM method is being used to manufacture tooling for pressure casting moulds [10]. The SLM method makes it possible to produce complex parts with optimised geometries while using materials efficiently and in a relatively quick and simple manner. However, the design of reliable parts requires us to identify the changes that this technology brings in terms of material properties and the impact of typical defects as compared to conventional manufacturing methods. Defects that arise during the manufacture of parts by SLM can be classified as either external or internal. The most commonly observed external defects are delamination due to the bad recoating of the powder, part lifting from supporting structures or local delamination due to high thermal stresses, surface distortion due to poor thermal conductivity, excessive down-facing roughness, and slippage defects due to stepwise positioning errors between layers [11]. Surface defects affect the fatigue strengths of parts that are made using the SLM method; however, these are eliminated by machining in many cases (which is necessary due to the excessive surface roughness of the printed part). For those parts of the mould that reproduce the shape of the casting, machining is essential. The most common internal defects in material after the SLM process are keyhole pores that are caused by gas that is trapped inside the powder during the gas atomisation process or gas that is generated in the molten pool due to the high solubility of the interstitial elements in the liquid phase coupled with rapid solidification. Unmelted powder exists in the central area of the molten pool, and small cavities form due to inadequate processing parameters, leading to the improper solidification of the material before it is completely fused to the other parts [12]. Internal defects affect the strength of the material and the eventual crack propagation following the initiation phase. In addition to the tests that were carried out on the specimens in the present study (which resulted in the determination of the reference strength properties of the material), the strength of a mould core model that was made using the SLM method was also tested. The aim of these tests was to compare the reference properties of 1.2709 SLM steel with the strength and hardness of the core material.

A mould is subject to cyclical temperature changes that are caused by successive phases of the pressure machine cycle. Large changes in mould surface temperatures are caused by contact with liquid metal, heat dissipation during the solidification of a casting, opening the mould, removing castings, and spraying liquid on the mould’s surface before it is closed again. Temperature changes generate a thermal and mechanical stress field in the mould, resulting in the phenomenon of the thermo-mechanical fatigue of the mould material [13,14,15,16]. This phenomenon is particularly intense in the areas of the mould or core that are in contact with the liquid metal as well as in the vicinity of the surfaces of the cooling channels. During pressure machine cycles, mainly compressive stresses occur on the mould surface in contact with the metal, while tensile stresses dominate on the channel surfaces. The occurrence of tensile stresses on the surface of the channels causes a high risk of crack initiation and propagation in this zone of the mould. This fact inspired the article’s authors to undertake the research, the results of which are presented in this article. The duration of the crack initiation phase largely depends on the surface roughness and (to a lesser extent) on defects within the material that are created during the printing [17,18]. The surfaces of the mould or core that are in contact with the metal are always machined, whereas the surfaces of the cooling channels in the SLM mould core are not machined. Their surface has the texture and roughness produced by the SLM process. In contrast to the external surfaces of a mould cavity or core, the initiation of a crack on a channel surface does not immediately eliminate the mould from use. Crack development is highly dependent on the values of the components of the stress–strain tensor, as well as on the triaxiality factor. Therefore, this article proposes a plasticity loss criterion based on the analysis of the stress–strain state. This criterion can be used to predict the possibility of cracks forming on the surface of mould cooling channels. Due to the much higher operating temperature range of a pressure mould compared to an injection mould, the use of conformal cooling poses greater research and design problems. Consequently, the use of the right steel grade, SLM process parameters, and heat treatment parameters is a very important issue. The most commonly used material for SLM mould parts is maraging steel powder with the symbol of 1.2709. The SLM process results in a steel with a martensitic structure whose hardness is too low as related to that which is required for pressure mould parts. Therefore, heat treatment is recommended for the printed parts, which results in the strengthening of the alloy through the separation of the intermetallic phases [19,20,21]. The recommended ageing process for 1.2709 SLM steel is carried out at 490 °C [22] or 540 °C [23], obtaining hardnesses of 50–57 and 52 HRC, respectively. An increase in the ageing temperature results in austenite reversion, decreases in toughness and hardness, and a concomitant increase in the ductile properties of the steel [24]. The choice of heat treatment parameters for a pressure mould part should ensure that a material with the required hardness, a high yield strength, and sufficient ductility is obtained. As mentioned above, a characteristic feature of the conformal channel is the frequent change in its trajectory, resulting in numerous surface kinks in the forms of curves with small, rounded radii. As a result, a notch-influenced phenomenon occurs in numerous places in the area in which a triaxial stress state is present. The triaxial stress state limits the development of plastic deformation as the stress level increases. This phenomenon means that a material that exhibits plastic characteristics in a static tensile test can change its properties and become brittle. In the presence of material defects in the forms of inclusions, voids, or unmelted powder grains, the triaxial stress state stimulates the formation of microcracks in their surroundings, which increase in size due to plastic deformation until the voids coalesce and eventual plastic fracturing occurs. This paper presents the results of an experimental study to determine the effect of changes in triaxiality factor η on the value of equivalent strain ε_eq_ for maraging steel that was obtained by the SLM method and subjected to two types of heat treatment. Experimental results and FEM calculations were used to determine the plasticity loss of SLM steel on the surface of the conformal cooling channel based on the proposed criterion. 

## 2. Materials and Methods

The test specimens and mould core model were made by the SLM method from maraging steel powder with the trade symbol of “CL50WS” and obtained by gas atomisation (recommended by Concept Laser General Electric Additive Company). The chemical composition of the powder corresponds to martensitic, hot work steel 1.2709. The chemical composition of the powder (determined on the basis of spectroscopic tests) is listed in Table 1.

The chemical composition of the powder was determined by spectral analysis using a Spectro Midex energy-dispersive X-ray fluorescence spectrometer (ED-XRF) from SPECTRO Analytical Instruments GmbH, Kleve, Germany. The samples for the testing and the core were made using an M2 Concept Laser Cusing device. The material was printed with the parameters that are listed in Table 2. The sample and core axes were located perpendicular to the surface of the M2 printer work platform.

**Figure 1:**
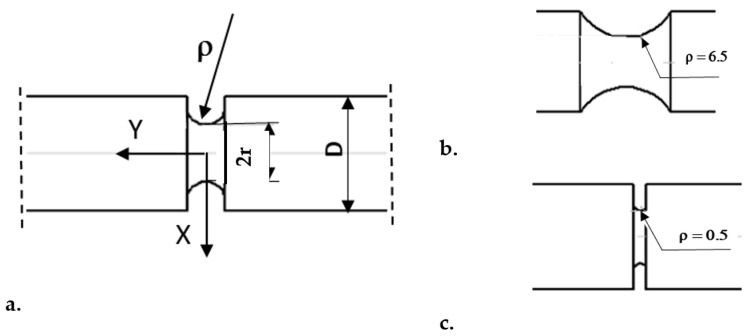
Circumferentially notched specimen for tensile tests in triaxial stress state: (**a**)—letter designation of main dimensions; (**b**)—notch ρ = 6.5 mm; (**c**)—notch ρ = 0.5 mm.

The island-scanning pattern was used. In this scanning strategy, part of the surface of each slice was divided into small square islands. The islands were scanned in a random manner, while the scanning direction was altered at right angles with respect to the neighbouring islands. The relative density of the fabricated specimens was measured according to Archimedes’ principle. The relative density of the fabricated specimens reached 99.91%. Smooth specimens were not machined. The surface of the notches has been machined to ensure dimensional accuracy. The material was heat treated by ageing at temperatures of 490 °C (HT490) and 540 °C (HT540) for 8 h. The heating and cooling of the specimens was carried out at a rate of 100 K/h. Heat treatment was carried out in a “Mini Tube KJ 1200” (Zhengzhou Kejia Furnace Co., Ltd., Zhengzhou, China) oven. The diameter of the furnace chamber was 40 mm, and its length was 200 mm. The control and steering of the temperature changes was carried out by a microprocessor. Uniaxial tensile tests were carried out on specimens with a diameter of 5 mm using an MTS 810 testing machine according to ISO 6892-1. An MTS extensometer with a 20-mm base was used to measure the elongation. The specimens that were intended to be subjected to investigations in a multiaxial stress state had circumferential notches with different radii of ρ = 0.5, 1.5, 2.5, 3.5, 4.5, and 6.5 mm (as shown in Figure 1). The diameter of all of the specimens was D = 10 mm, and the minimal diameter at the notch was 2r = 5 mm. The strain rate for the smooth specimens was 0.001 s^−1^ in the elastic range and the predicted strain rate was 0.002 s^−1^ when the yield point was exceeded. For notched specimens, the test stress rate was 2.5 MPa/s. The number of smooth specimens was 5 for each heat treatment (10 specimens in total), whereas ring notched specimens were 4 for each notch radius (48 in total).

Measurement of the diameter of the specimen at the bottom of circumferential notch 2r and the radius of notch ρ before and after the tensile test (2r_0_ and ρ_0_ respectively) were carried out using an optical microscope image and a clamping fixture that was specially made for this purpose. The metallographic tests of the microsections and fractures were performed using a Nikon SMZ 745T stereoscopic optical microscope (Konan, Japan), a Nikon Eclipse metallographic microscope with a DsFi1 camera that enabled digital image analysis, and a Hitachi S-3400N scanning electron microscope (Tokyo, Japan) with a Thermo Noran energy dispersive X-ray spectroscope (Waltham, MA, USA). Strength tests of the core that was made by the SLM method were carried out according to the following procedure using DIN SPEC 4864: “Test method for the determination of flow curves and benchmark characteristic values for tensile testing by means of minor destructive indentation, 3D measurement, and finite-element material models” [25]. The numerical calculations that were used to determine the strain field and triaxiality factor η in the mould core were performed with the program Abaqus v. 2019.

## 3. Thermal and Strain–Stress Model of Phenomenon

The High Pressure Die Casting (HPDC) process repeats cyclically. In the numerical model, the duration of one HPDC cycle was assumed to be 35 s. The thermal model assumed the division of each work cycle into four stages: alloy crystallisation and casting cooling—22 s; casting extraction—2 s; spraying the surface of the mould with water suspension—3 s; and preparing the mould for reclosure—8 s. The cycle time assumptions given are based on the operating parameters of the FRECH 510 horizontal cold-chamber HPDC machine. The core with the conformal cooling channel referred to in the article is mounted in the mould and works in the cycle described.

**Figure 2:**
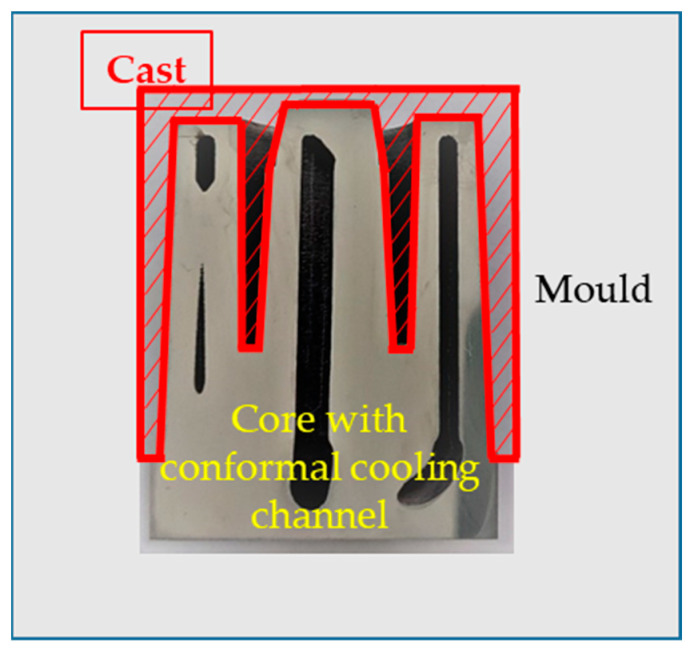
Position of mould core, mould, and casting in geometrical model.

The geometrical model consisted of a core, casting, and mould cavity; all are shown in Figure 2. A thermal surface contact model was used to describe the boundary conditions of the heat transfer between the mould and the casting. In the model, the temperature-dependent properties of the AlSi9Cu3 alloy were assumed. The calculations assumed an initial alloy temperature of Tinit_1 = 680 °C and an initial core temperature of Tinit_2 = 50 °C. 

The strain–stress model took the geometry of the casting and the conformal core into account. Its attachment was determined by taking away degrees of freedom on the mould’s contact surfaces. The strains and stresses and the triaxiality factor η in the core were calculated based on the temperature fields. As a result of numerical calculations, the following were calculated: the values of equivalent deformation ε_eq_ as determined by Equation (1) [26], and triaxiality factor η (whose definitions and discussion are presented in Section 3.1).

(1) $$\varepsilon_{eq}=\frac{1}{\left(1+\nu \right)\sqrt{2}}\sqrt{{\left(\varepsilon_{1}-\varepsilon_{2}\right)}^{2}+{\left(\varepsilon_{2}-\varepsilon_{3}\right)}^{2}+{\left(\varepsilon_{3}-\varepsilon_{1}\right)}^{2}},$$

where ε_1_, ε_2_, ε_3_ are the principal strains, and ν is Poisson’s ratio.

A detailed description of the temperature model and the material characteristics that were adopted in the numerical calculations are presented in [27].

### 3.1. Criterion for Plasticity Loss

The material resistance is usually estimated during the uniaxial tensile test (even though the material is usually subjected to multiaxial stress states in many parts of the structure). In a multi-axis stress state, the properties of the material and the manner of its decohesion change with the value of triaxiality factor η. The triaxiality factor is defined as the ratio of mean stress σ_m_ to the Mises criterion σ_v_ [28,29].

(2) $$\eta =\frac{\sigma_{m}}{\sigma_{V}},$$

(3) $$\sigma_{m}=\frac{\sigma_{1}+\sigma_{2}+\sigma_{3}}{3},$$

(4) $$\sigma_{V}=\frac{1}{\sqrt{2}}{\left[{\left(\sigma_{1}-\sigma_{2}\right)}^{2}+{\left(\sigma_{2}-\sigma_{3}\right)}^{2}+{\left(\sigma_{3}-\sigma_{1}\right)}^{2}\right]}^{\frac{1}{2}},$$

where σ_1_, σ_2_, and σ_3_ denote the principal stresses.

Triaxiality factor η can often be used to predict the type of fracture within a region that is defined by this stress state. High-stress triaxiality promotes brittle cleavage fractures as well as dimple formation within an otherwise ductile fracture. Low-stress triaxiality corresponds with shear slip and, therefore, greater ductility. An experimental observation of a triaxiality factor that is greater than 2/3 rather indicates that the biaxiality condition of the plane stress test was lost and a three-dimensional stress state started to exist in the sample [30]. Experimental studies to determine the effect of triaxiality factor η on changes in the plastic properties of the material can be carried out on circumferentially notched specimens. The stress distribution in the region of a notch that is made in the bar can be determined by using the Bridgman equation [31,32]. This equation gives the relationship between triaxiality factor η at the point of intersection of the specimen axis with the plane that passes through the bottom of the circumferential notch:

(5) $$\eta =\frac{1}{3}+\ln\left(1+\frac{r}{2\rho }\right)$$

The values of the η coefficients for the circumferential notch samples with different radii ρ were calculated based on Equation (5). The radius of ring notch ρ was determined for each sample based on the image that was obtained by the optical microscope; they were measured in the same way. The equivalent strain ε_eq_ along the minimum cross section is given by the following [33]:

(6) $$\varepsilon_{eq}=2\ln\left(\frac{r_{o}}{r}\right)$$

The η values that occurred in the pressure mould core model during the operating cycle were determined from the numerically calculated components of the stress tensor using Equations (2)–(4). Based on the experimental results, the graphs of function ε_eq_/ε_eq_s_ = f(η) were constructed (ε_eq_s_—the equivalent strain of the smooth sample at rupture). Function f(η) was proposed as a criterion for the plasticity loss of the material in the area of the pressure mould cooling channels.

## 4. Results

### 4.1. Strain and Triaxiality Factor in Mould Core Model

**Figure 3:**
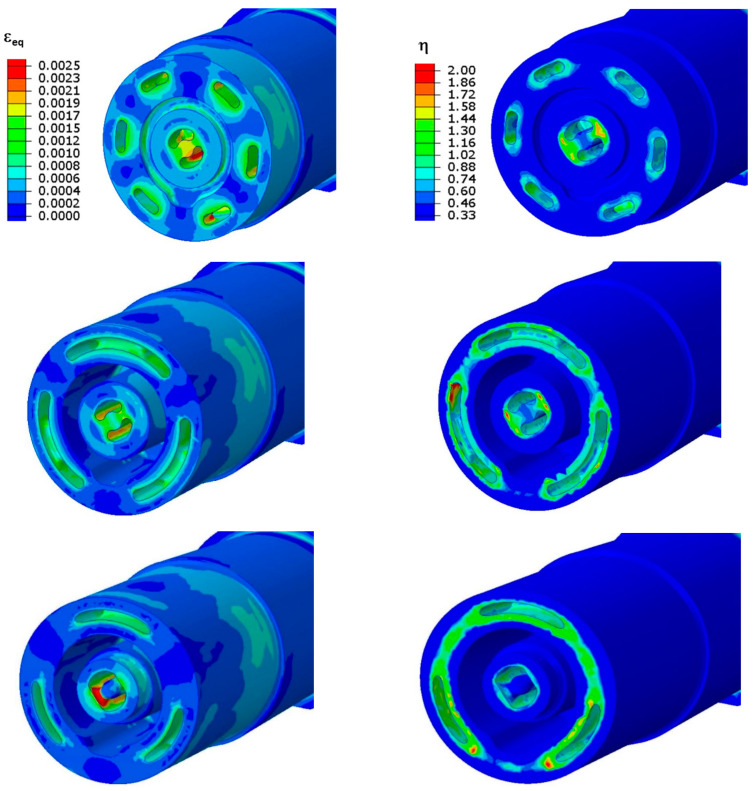
Field values of equivalent strain ε_eq_ (**left** column) and triaxiality factor η (**right** column) shown in three cross sections of core with conformal cooling channel approximately two seconds after metal was injected into mould.

**Figure 4:**
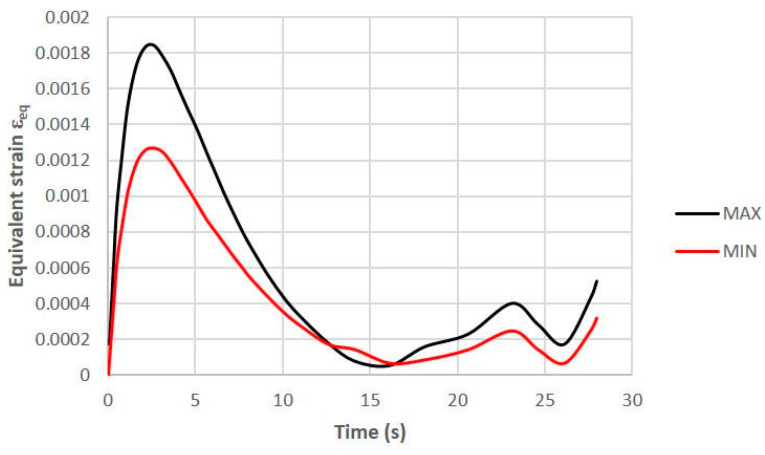
Typical course of changes in value of equivalent strain ε_eq_ in area of change in trajectory of axis of cooling channel (“MAX” curve) and in vicinity of linear channel (“MIN”) curve that occurred during one cycle of pressure mould operation.

As a result of the FEM calculations, the changes in the equivalent strain ε_eq_ and triaxiality factor η that occurred in the core during the stabilised cycle of the pressure mould were determined. In Figure 3, the left column shows the field of equivalent strain ε_eq_ in three sections of the core after 2.1 s from the moment of injecting the metal into the mould (when their values were the highest), while its right column shows the field of the triaxiality factor η values in the same sections of the core and at the same moment of time. Both the strain and the triaxiality factor had the highest values in the vicinity of the cooling channels. Most often, the value of equivalent strain ε_eq_ in the vicinity of the channels is between 0.0012 and 0.0015 (mm/mm); however, the maximum value of the strain increased to about 0.0020 (mm/mm) in the area where there was a large curvature of the channel surface (the area where the direction of the trajectory of the cooling channel axis changed). A typical course of changes in the ε_eq_ strain values during the pressure mould cycle in the area of the change in the trajectory of the channel axis (notch effect) is shown by the “MAX” curve in Figure 4, while the “MIN” curve is shown in the surroundings of the linear channel. Figure 4 shows the initial rapid increase in the strain immediately following the injection of the metal into the mould. The effect of the rapid increase in temperature and the associated increase in the strain values was enhanced by the relatively short distance of the cooling channel from the core–metal contact surface (which was about 4 mm).

### 4.2. Test Results of 1.2709 SLM Steel in Triaxial Stress State

#### 4.2.1. Tensile Tests

Strength tests were carried out on smooth specimens in uniaxial stress and triaxial stress states using circumferentially notched specimens. As the results of the uniaxial tensile test, the ultimate tensile stress (UTS), yield stress (σ_p0.2_), modulus of elasticity (E), and elongation (A) were obtained. The results of the tensile test for two variants of heat treatment (“HT490” and “HT540”) are listed in Table 3 as the results that were obtained on the four specimens.

**Figure 5:**
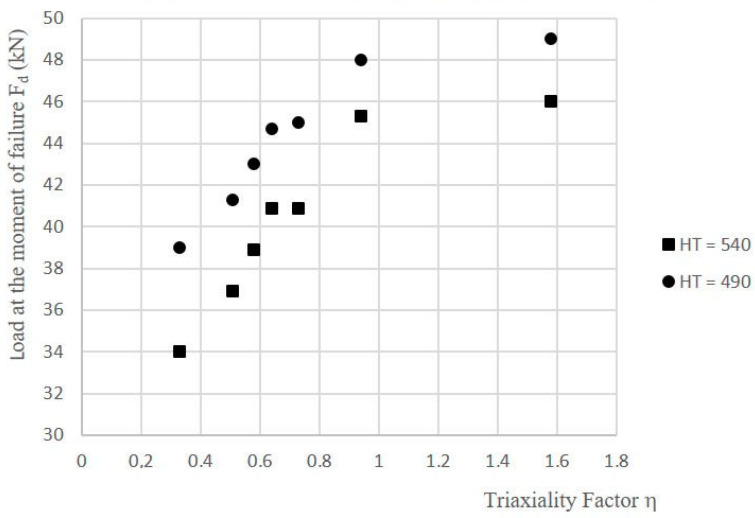
Influence of triaxiality factor η on value of destructive force F_d_ of 1.2709 SLM steel subjected to two heat-treatment variants (HT490 and HT540).

**Figure 6:**
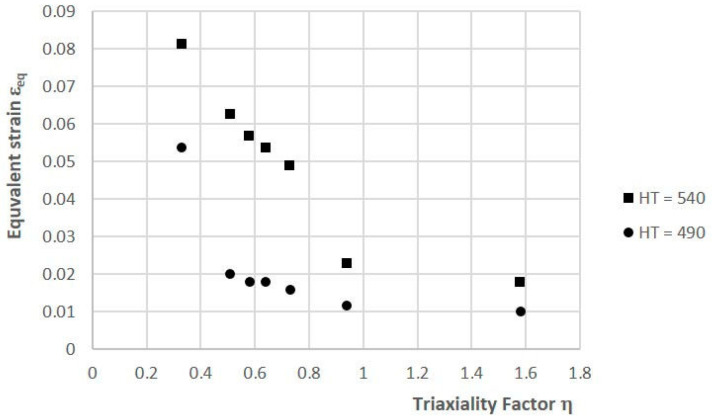
Influence of stress triaxiality factor η on equivalent strain ε_eq_ of 1.2709 SLM steel subjected to two heat-treatment variants (HT490 and HT540).

According to the study, a higher ageing temperature causes a decrease in the strength of SLM steel as well as a simultaneous increase in its ductile properties. The tests that were conducted on circumferentially notched specimens in a triaxial state of stress showed a very large effect of triaxiality factor η on the strength and ductility of the steel. As η increased, the value of the destructive force of specimen F_d_ increased (Figure 5), while the value of the equivalent strain at the bottom of notch ε_eq_ decreased (Figure 6).

#### 4.2.2. Fractographic Studies

**Figure 7:**
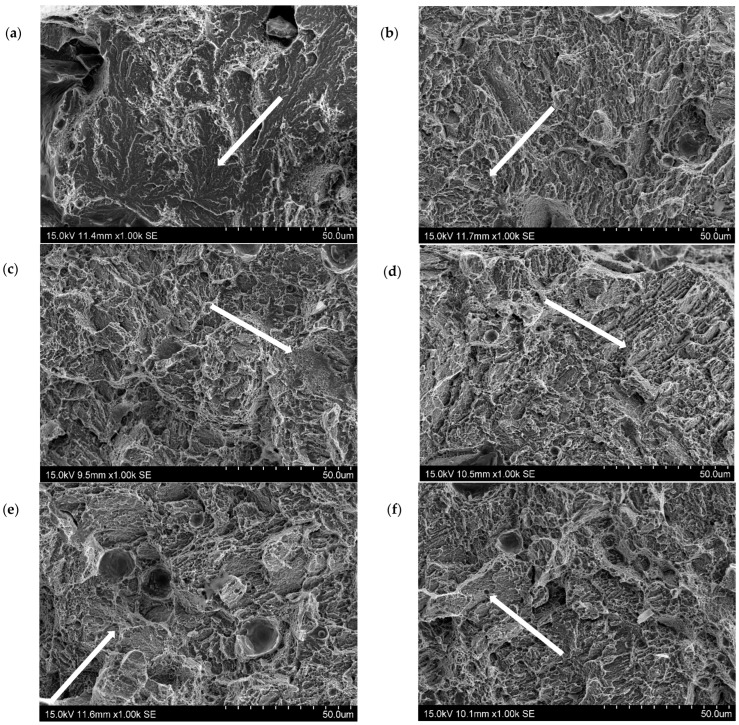
SEM images of fractures of circumferentially notched specimens made by SLM method of 1.2709 steel powder after HT490 heat treatment—triaxiality factor values: (**a**) 1.58; (**b**) 0.94; (**c**) 0.73; (**d**) 0.64; (**e**) 0.58; (**f**) 0.51; white arrows indicate areas of brittle fracture.

The main aim of the fractographic examination was to make a qualitative assessment of the fracture morphology in relation to the stress triaxiality factor. The examinations were made on all of the specimens and tested under different spatial stress states. Several photos were taken at different magnifications of the microregions in the centre of each sample. In the central part of the specimen, the hydrostatic stresses were the highest; in this area, triaxiality factor η had the highest value when the specimen ruptured. These examinations indicated that the stress state influenced the fracture surface morphology. Figure 7 shows typical fracture images of specimens that were made from 1.2709 steel powder by SLM and aged at 490 °C. The images show the fractures of six specimens with different triaxiality factors.

**Figure 8:**
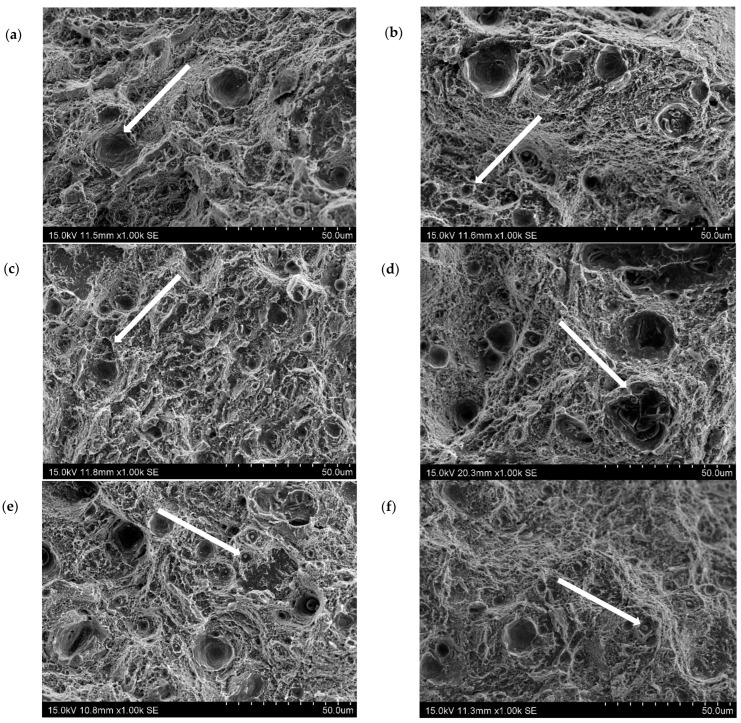
SEM images of fractures of circumferentially notched specimens made by SLM method of 1.2709 steel powder after HT540 heat treatment—triaxiality factor values: (**a**) 1.58; (**b**) 0.94; (**c**) 0.73; (**d**) 0.64; (**e**) 0.58; (**f**) 0.51; white arrows indicate where dimple holes are present at the fracture.

The circumferentially notched HT490 samples with triaxiality factors η = 0.51, 0.58, 0.73, and 0.94 that showed brittle fractures are visible in Figure 7a–d. The fracture images of the samples with triaxiality factors η = 0.58 and 0.51 (Figure 7e,f) showed areas where plastic fracture features could be identified in the form of characteristic dimples. Images of the fracture surfaces of the HT540 heat-treated specimens showed areas that exhibited features of both brittle and plastic fracture regardless of the value of η (Figure 8).

Circumferentially notched specimens with notch radii of ρ = 0.5 and 1 mm (Figure 8a,b) had a distinguishably higher proportion of brittle fractures than the others (Figure 8c–f). The observations of the fractures correlated with the results of the strength tests, which showed decreases in the proportions of the ductile properties of the SLM steels with increases in η and as a result of the heat treatment at the lower temperature.

## 5. Results of Study of Chemical Composition of Conformal Core

**Figure 9:**
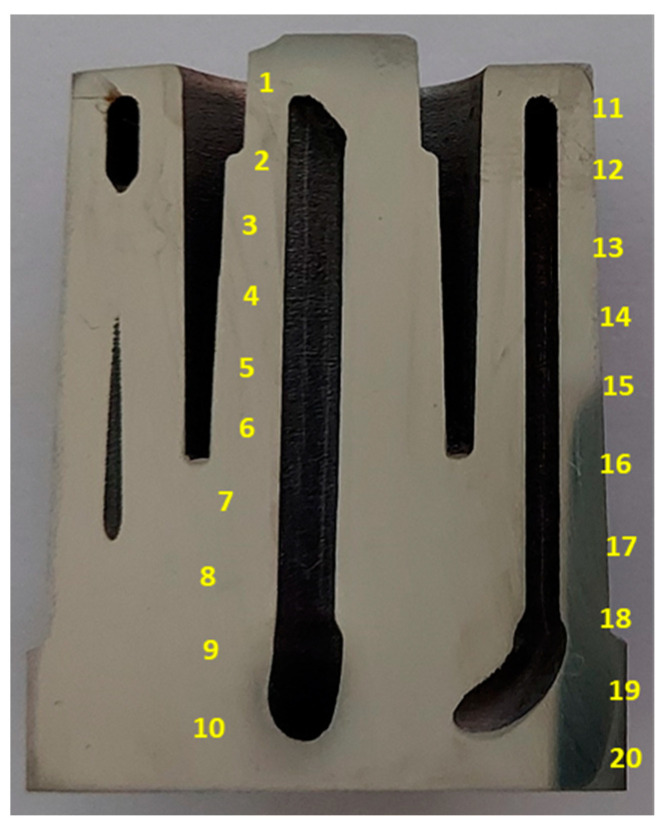
Locations of measurement points on surface of core section where chemical composition of SLM 1.2709 steel was determined.

The printed 1:2 scale model of the core was cut into two parts. Chemical composition tests were carried out at 20 measurement points on the surface that resulted from the cutting of the core (Figure 9). Cutting the core revealed the outlines of a conformal cooling channel (parts of which can be seen in Figure 9). Table 4 shows the results of the chemical composition tests on the surface of the core section and, additionally, gives the maximum (Max) and minimum (Min) values of the results that were obtained as well as the mean values for the 20 measurements and the standard deviations (σ). The elemental percentages were within the ranges of the values that were expected for this steel grade with the exception of Mo (4.37%) and Ti (0.77%); these average percentages were below the lower limits of the required ranges of the values of Mo (4.50–5.20%) and Ti (0.80–1.20%). The relatively large local fluctuations in the percentage of Ti could have been caused by the formation of large oxide precipitates of this element of about 20 µm, which significantly reduced the mechanical properties of the SLM steels [34].

## 6. Test Results for Mechanical Properties of Mould Core

**Figure 10:**
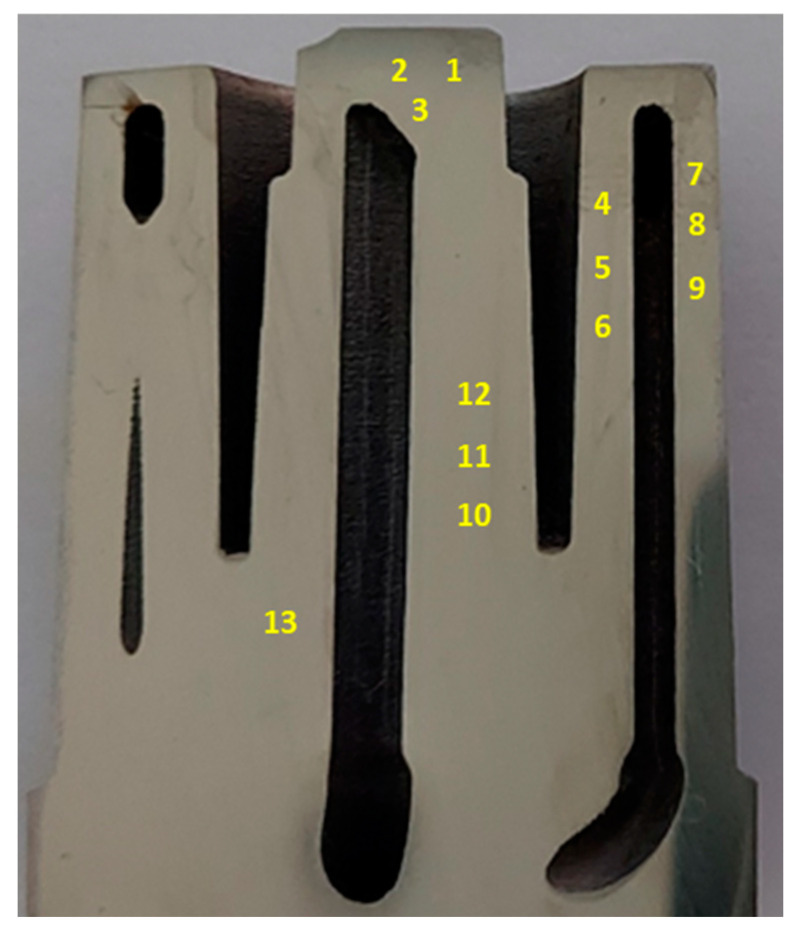
Measurement points of yield strength (R_p0.2_, R_p1.0_), ultimate tensile strength (UTS), and plastic strain (ε_pl_) marked on cross-sectional surface of pressure mould core.

**Figure 11:**
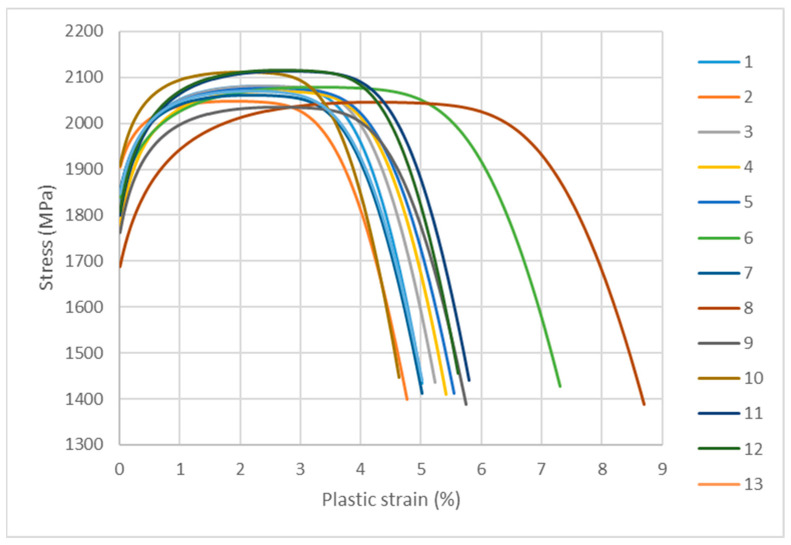
Plots of stress–strain function defined at Points 1 through 13 (labelled in Figure 10); test was performed in accordance with DIN SPEC 4864:2019-11 standard.

The testing of the mechanical properties of the pressure mould core was carried out according to DIN SPEC 4864:2019-11 on the same cross-sectional area as for the chemical composition tests. The measurement locations were marked as shown in Figure 10. The method consisted of making a dimple in the test material through an indenter, which was moved orthogonally to the sample surface and penetrated the sample in a force-controlled manner until the test force value was reached. After a specified amount of time, the indenter was removed from the sample. An optical measuring device then determined the resulting three-dimensional deformation of the sample. A finite element programme created a simulation model that provided a realistic representation of the hardness test (constitutive material behaviour, mechanical boundary conditions, friction). The programme’s algorithm changed and adjusted the material properties until the shape and dimensions of the virtual dimple (with sufficient accuracy) corresponded to the image of the actual dimple that was obtained by the optical measuring device. The optimisation algorithm changes the coefficients describing the stress–strain curve and simulates, using the FEM method, a virtual indentation imprint in the test material. The coordinates of the points on the surface of the virtual indentation are compared with the actual coordinates of the points in the optical image. If the spatial mapping is optimised, the difference between the virtual and actual coordinates of the indentation surface cannot be greater than 5%, if the mapping is axisymmetric 3%. The mechanical properties of the materials that were tested were taken from the simulation with the best match between the FE solution and the actual shape and dimensions of the dimple. The results of the tests that were carried out are presented in the forms of graphs as functions of the plastic stress strain (Figure 11). These graphs are similar to those that were obtained in the classical tensile test of the metals; the difference lies in the absence of the elastic strain part of the graph, which was not determined due to the nature of the method. Table 5 summarises the results of the yield strength (R_p0.2_ and R_p1.0_), ultimate tensile stress (UTS), and plastic strain (ε_pl_) as determined from the graphs that are presented in Figure 11.

Table 5 shows the results of the strength tests that were carried out on the surface of the mould core section in accordance with DIN SPEC 4864:2019-11. Table 5 also shows the maximum (MAX) and minimum (MIN) values of the results that were obtained as well as the mean values and standard deviations (σ) and the differences between the maximum and minimum values (Δ = Max–Min). 

**Figure 12:**
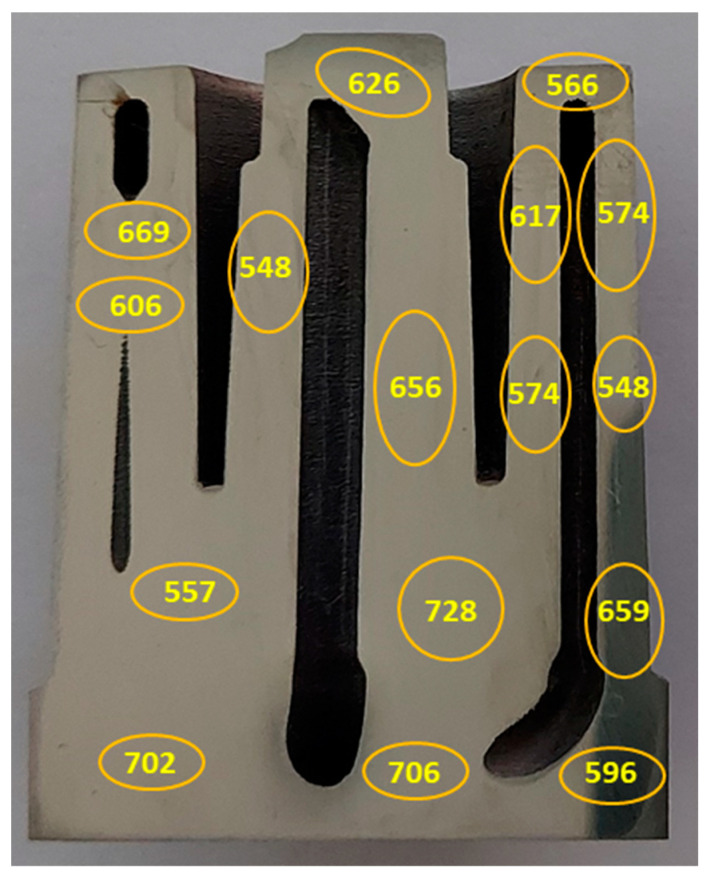
Results of HV microhardness measurements carried out on surface of conformal section of mould core.

Microhardness measurements were also taken on the cross-sectional surface of the core; it was discovered that there were significant differences between the values at different points. The results of these measurements confirmed the variations of the mechanical properties within the mould core that was made by the SLM method. Figure 12 shows the average values from several microhardness measurements that were carried out in the areas that were marked with an ellipse on the cross-sectional surface of the core.

## 7. Analysis of Results

**Figure 13:**
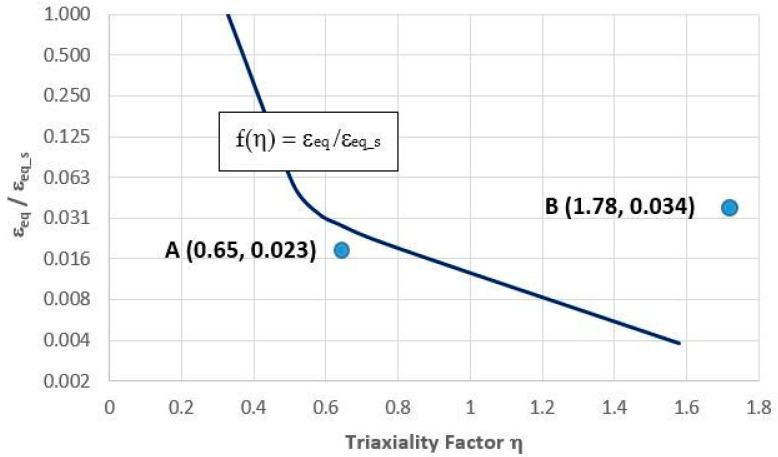
Locations of Points A and B with coordinates defined by strain quotient ε_eq_/ε_eq_s_ and value of triaxiality factor η determined by FEM method during one cycle of pressure machine against experimentally determined function f(η) = ε_eq_/ε_eq_s_ for SLM steel after HT490 heat treatment: Point A—value of function in vicinity of rectilinear channel; Point B—value of function in vicinity of channel with curvilinear axis trajectory.

**Figure 14:**
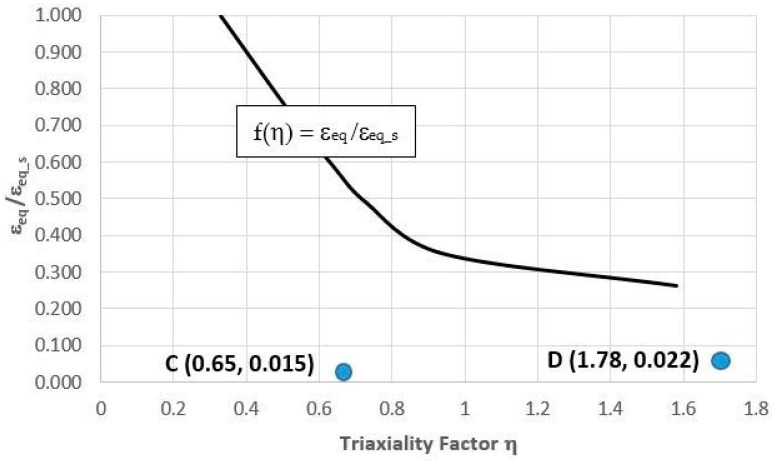
Locations of Points C and D with coordinates defined by strain quotient ε_eq_/ε_eq_s_ and value of triaxiality factor η determined by FEM method during one cycle of pressure machine against experimentally determined function f(η) = ε_eq_/ε_eq_s_ for SLM steel after HT540 heat treatment: Point C—value of function in vicinity of rectilinear channel; Point D—value of function in vicinity of channel with curvilinear axis trajectory.

The experimental studies and numerical simulations that are presented in this paper were carried out to determine the plasticity loss criterion for steel 1.2709 that was obtained by SLM and then subjected to different heat treatment variants. Based on the formulated criterion, it was possible to determine the allowable value of equivalent strain ε_eq_ in the insert or core of the pressure mould as a function of the degree of triaxiality factor η. The criterion can be used in the vicinity of cooling channels, as the value of the mechanical strain there has a direct influence on the strength of the material. The criterion loses validity in those areas of the mould part where the influence of thermal deformation cannot be neglected. The use of the plasticity loss criterion to determine the allowable values of mechanical strains as a function of changes in the triaxiality factor in a core is justified due to the frequently occurring changes in the direction of the axis and the conformal cross section of the cooling channel. Changes in the trajectory of the channel axis cause a notch effect and, thus, a local increase in the triaxiality factor. The result is a reduction in the ductile properties of SLM steels in favour of an increase in strength. Steel whose fracture in the uniaxial stress state has plastic characteristics in the triaxial stress state may have a brittle fracture. This criterion was defined in the form of the function f(η) = ε_eq_/ε_eq_s_ that was determined from the results that were obtained from the tensile testing of the circumferentially notched specimens that were aged at 490 °C (Figure 13) and 540 °C (Figure 14). For the smooth unnotched specimens, the triaxiality factor η in the specimen axis that was calculated using Equation (5) was 0.33, and the value of function f(η) = 1. Figure 13 shows Points A and B with coordinates (ε_eq_/ε_eq_s_, η). Equivalent strain values ε_eq_ and η in the mould core during one cycle of the pressure machine were determined using the FEM method. The coordinates of Point A were determined by the maximum values of ε_eq_ and η that occurred in the vicinity of the rectilinear sections of the cooling channel, while the coordinates of Point B were determined by the maximum values of ε_eq_ and η that were in the vicinity of the curved sections. The ε_eq_s_ strain was the strain of the smooth specimens that were aged at 490 °C. The experimental tests and numerical calculations showed that the HT490 heat treatment caused such a large loss of plasticity in the 1.2709 SLM steel that the ε_eq_ strain and η values that were determined in the core exceeded permissible values (Figure 13).

The failure mode was studied using a scanning microscope on the fracture surfaces of the samples. The specimens were observed and photographed at a central area of the fractured cross section (thus, on the spot that corresponded to the maximal stress concentration). The plasticity loss that was caused by the heat treatment that was shown in the tensile tests was confirmed by the fractographies. The ductile failure of the SLM steel took place through the initiation, increase, and coalescence of the voids that were generated on the inclusion. The voids that were generated around the inclusion during the loading had their reflections as dimples on the fracture surface; this type of fracture can be seen in Figure 7e,f and Figure 8a–f. The brittle failure nucleation locations were the same as the ductile fracture, but the fracture mode was different. The brittle fracture was characterised by a very small proportion of plastic deformation and was mainly developed by cleavage decohesion; this type of fracture is characteristic of heat treatment at 490 °C (Figure 7). In the same way as for Points A and B, the coordinates of Points C and D in Figure 14 were determined. The positions of Points C and D with respect to function f(η) = ε_eq_/ε_eq_s_ indicates that the heat treatment of HT540 caused such a sufficiently small decrease in the ductility of the 1.2709 SLM steel that the values of the strain and triaxial factor in the vicinity of the cooling channels did not exceed permissible values. The numerical calculations showed that the values of η in the zone of the rectilinear channels (η = 0.65) differed significantly from those in the areas where the curvature of the channel axis changed (η = 1.78). These values changed only slightly during the pressure mould cycle despite the significant changes in the stress and strain (Figure 4). This, of course, applies to the stabilised mould cycle, which occurred several cycles after the first metal injection.

**Figure 15:**
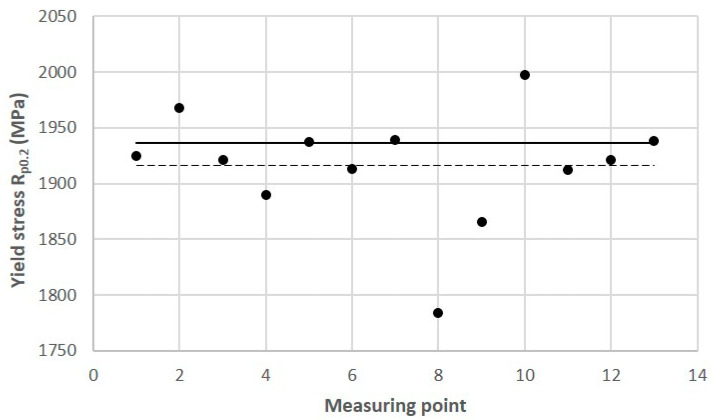
Yield strength values (R_p0.2_) determined at measurement Points 1 through 13 on cross-sectional area of mould core (Figure 10) according to DIN SPEC 4864:2019-11: solid line—reference yield strength value R_p0.2_ = 1936 MPa (determined in static tensile test); dashed line—average value of R_p0.2_ = 1916 MPa for Points 1 through 13.

**Figure 16:**
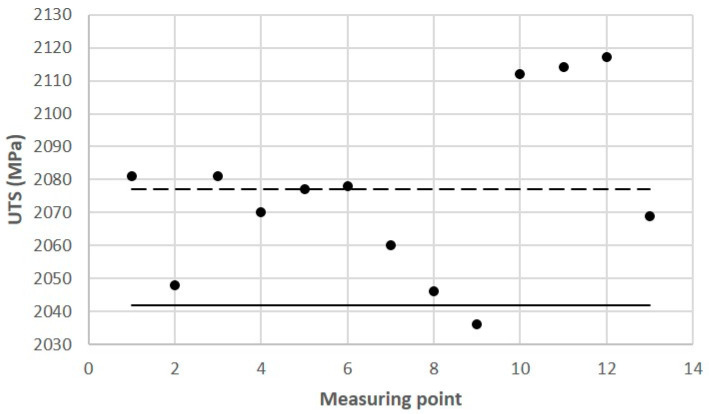
Values of ultimate tensile strength (UTS) determined at measurement Points 1 through 13 on cross-sectional area of core (Figure 10) according to DIN SPEC 4864:2019-11: solid line—reference value of UTS = 2042 MPa (determined in static tensile test); dashed line—average value of UTS = 2076 MPa for Points 1 through 13.

**Figure 17:**
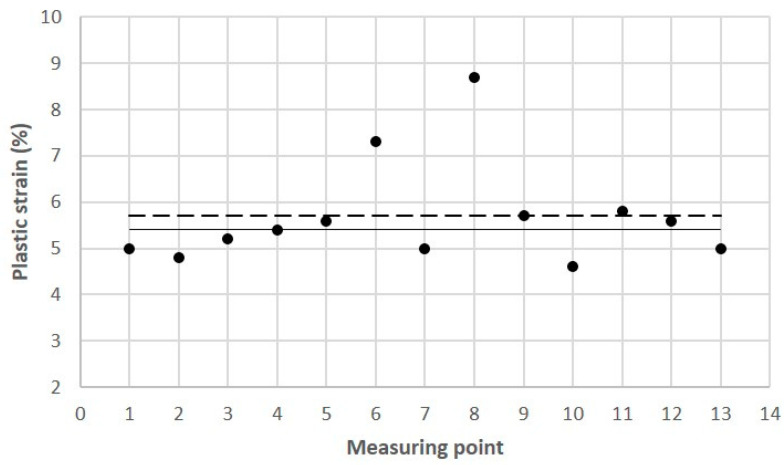
Plastic strain values (ε_pl_) determined at Measurement Points 1 through 13 on cross-sectional area of core (Figure 10) according to DIN SPEC 4864:2019-11: solid line–reference plastic strain value ε_pl_ = 5.41% (determined in static tensile test); dashed line–average value of ε_pl_ = 5.70% for Points 1 through 13.

Tests of some of the mechanical properties of the 1.2709 SLM steel were carried out directly on the cross-sectional surface of the printed core in order to compare the results with the reference values that were determined in the tensile and microhardness tests. The SLM process parameters were the same during the printing of the core model and the samples. It was found that the values of yield strength R_p0.2_, ultimate tensile strength UTS, and the plastic strain of the SLM steel differed depending on the position of the measurement point on the cross-sectional surface of the core. Figure 15 shows the yield strength values of R_p0.2_ measured at the 13 points as related to their mean value (represented by the dashed line) and to the value of R_p0.2_ as determined in the static tensile test (solid line). The difference between the largest and smallest values of R_p0.2_ that were measured on the cross-sectional area of the core was as high as 213 MPa, and the standard deviation from the mean value for the population of the 13 measurement points was σ = 49.2 MPa. Much smaller was the difference between the average value of R_p0.2_ that was determined at the same measurement points and the yield strength R_p0.2_ that was determined in the tensile test—this was 20 MPa. A similar graph was made for the measured UTS values (Figure 16). The difference between the largest and smallest UTS value was 81 MPa, and the standard deviation was σ = 25 MPa. The difference between the average UTS value that was determined for the 13 measurement points and the UTS strength that was determined in the tensile test was 35 MPa in this case. When performing a strength test according to DIN SPEC 4864:2019-11, only the plastic strain of the material’s ε_pl_ is determined (the elastic part of strain ε_s_ is omitted). For this reason, Figure 17 shows the plastic strain value that was determined from the static tensile test plots of the SLM steel after omitting the elastic strain. The difference between the largest and smallest ε_pl_ values was 4.1%, and the standard deviation was σ = 1.1%. The difference between the mean value ε_pl_ that was determined at the measurement points and the plastic deformation of the steel that was determined in the tensile test was 0.52%. These results can be used to carry out an analysis to determine the appropriate safety factor for parts that are printed from 1.2709 steel powder via SLM.

**Figure 18:**
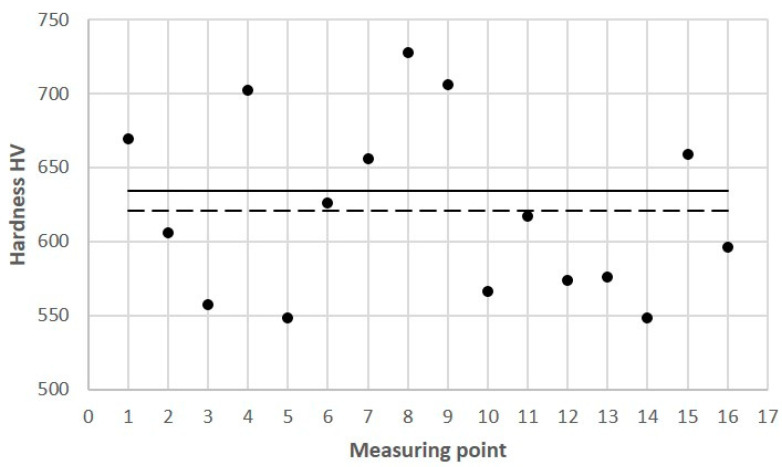
Microhardness values determined in 16 measurement areas on cross-sectional surface of mould core (Figure 12): solid line—hardness reference value of HV30 = 636 (determined on printed samples); dashed line—average microhardness value of 621 HV in 16 measurement areas.

As a supplement to the information on the differences between the reference mechanical properties of the SLM steels and those that were measured on the cross-sectional surface of the core, microhardness measurements were carried out on the cross-sectional surface of the core. Figure 18 shows the results of the measurements in the 16 areas of the core that were related to a reference value of 636 HV and an average value of the measurements of 621 HV at the 16 points. The standard deviation of the population of the core hardness measurements was 57.8 HV. The reference value was determined by taking a hardness measurement of HV30.

## 8. Conclusions

By using conformal cooling channels, it is possible to optimise heat exchange and transfer in the pressure mould. Mould parts cooled using conformal channels are made using the SLM method. Different from the traditional method of design, a conformal cooling channel system is characterised by a much smaller cross-sectional diameter of the channels, a much smaller distance between adjacent channels, and their smaller distance from the surface of the mould cavity (as well as numerous changes in the direction of the channel axes). All of the above-mentioned features of conformal channels cause a change and local increase in the components of the stress tensor, which can be determined by using triaxiality factor η. A local increase in the value of this factor causes the loss of plasticity in a material. For this reason, the paper proposes a so-called criterion for the loss of plasticity in steel, which can be used to assess a safe level for local strain and the value of triaxiality factor η in the zone of a mould’s cooling channels. Restricting the application of this criterion to the cooling channel zone only is related to the minimum value of the thermal deformation in this part of the mould or core. Based on the results of tensile tests on circumferentially notched specimens with different radii of rounding ρ and FEM calculations, a function f(η) was established that determines the allowable values of ε_eq_ and η. The measurements of the yield strength, plastic strain, and hardness that were carried out directly on the cross-sectional surface of the core showed differences as compared to the mechanical properties that were determined on the samples. This fact suggests the continuation and undertaking of new research and analysis that are aimed at determining safety factors for parts that are made via the SLM method. The plasticity loss method presented assumes that the value of the triaxiality factor in the vicinity of the channel is influenced by the shape, cross-sectional dimensions, and trajectory of the channel axis. Using the methodology proposed in this paper, it is possible to determine the value of allowable deformations in the cooling channel zone and to determine whether the heat treatment applied to the SLM steel does not cause an excessive reduction in the plastic properties. The scope of the applicability of the proposed criterion may not only be limited to the case of the core with a conformal channel that was analysed in this paper; it may also be applied where the so-called notch effect occurs and no thermal deformation is present.

## Figures and Tables

**Table 1:** Chemical composition; wt. % of powder.

Ni	Mo	Co	Ti	Cr	Fe
18.14	4.51	8.99	0.71	0.28	balance

**Table 2:** SLM process parameters for core and specimens.

SLM Parameter	Specimens	Core
Laser power (W)	180	380
Laser speed (mm/s)	1100	1500
Laser beam diameter (µm)	80	80
Layer thickness (µm)	25	40
Hatch space (µm)	90	90

**Table 3:** Results of uniaxial tensile and hardness tests.

Variant of Heat Treatment	σ_p0.2_(MPa)	UTS(MPa)	E(GPa)	A (%)	HV30/HRC
HT490	1936 (±11)	2042 (±5)	183 (±0.5)	7.31 (±0.5)	636/57
HT540	1696 (±10)	1743 (±6)	179 (±0.3)	11.98 (±0.6)	579/54

**Table 4:** Results of chemical composition tests carried out on surface of core section made from 1.2709 steel powder by SLM method.

Label	Ni	Co	Mo	Cr	Ti	Fe
1	17.950	9.043	4.331	0.208	0.760	66.830
2	18.110	9.033	4.393	0.216	0.769	66.860
3	18.000	9.049	4.312	0.204	0.783	66.840
4	18.190	9.023	4.377	0.206	0.782	66.860
5	18.030	9.004	4.414	0.215	0.830	66.710
6	18.130	9.041	4.357	0.211	0.820	66.810
7	18.180	8.969	4.419	0.199	0.842	66.800
8	18.080	9.014	4.376	0.214	0.850	66.650
9	18.180	9.019	4.411	0.218	0.850	66.530
10	18.190	9.021	4.332	0.208	0.833	66.710
11	18.120	9.035	4.352	0.213	0.751	66.710
12	18.100	8.999	4.347	0.226	0.721	66.800
13	18.200	9.074	4.352	0.207	0.697	66.900
14	18.120	9.082	4.355	0.196	0.749	66.910
15	18.000	9.088	4.322	0.208	0.780	66.810
16	18.200	9.026	4.436	0.208	0.746	66.790
17	18.140	9.055	4.378	0.206	0.729	66.650
18	18.140	9.071	4.465	0.218	0.700	66.680
19	18.230	9.086	4.334	0.244	0.720	66.640
20	18.220	9.066	4.407	0.224	0.745	66.650
Mean value	18.125	9.040	4.373	0.212	0.773	66.757
Max	18.230	9.088	4.465	0.244	0.850	66.910
Min	17.950	8.969	4.312	0.196	0.697	66.530
σ	0.077	0.031	0.040	0.010	0.049	0.100

**Table 5:** Values of yield strength (R_p0.2_, R_p1.0_), ultimate tensile strength (UTS), and maximum plastic strain (ε_pl_) determined on cross-sectional area of mould core.

Measuring Point	R_p0.2_ (MPa)	R_p1.0_ (MPa)	UTS (MPa)	ε_pl_ (%)
1	1925	2052	2081	5.0
2	1968	2037	2048	4.8
3	1921	2050	2081	5.2
4	1890	2032	2070	5.4
5	1937	2048	2077	5.6
6	1913	2026	2078	7.3
7	1939	2040	2060	5.0
8	1784	1944	2046	8.7
9	1866	1998	2036	5.7
10	1997	2096	2112	4.6
11	1912	2065	2114	5.8
12	1921	2071	2117	5.6
13	1938	2047	2069	5.0
Mean value	1916	2039	2076	5.7
Max	1997	2096	2117	8.7
Min	1784	1944	2036	4.6
σ	49.2	35.4	25.0	1.1
Δ	213	152	81	4.1

## Data Availability

The data that support the findings of this study are available from the corresponding authors, [J.P.; A.G.-K.], upon reasonable request.

## References

[1] Sefene E.M. (2022). State-of-the-art of selective laser melting process: A comprehensive review. J. Manuf. Syst..

[2] Phull G.S., Kumar S., Walia R.S. (2018). Conformal cooling for molds produced by additive manufacturing: A review. Int. J. Mech. Eng. Technol..

[3] Pancholi J.K., Padhiyar B., Sutariya V., Pancholi H. (2019). Design and Analysis of Die Casting Die with Conformal Cooling Channel. Int. J. Eng. Sci. Comput..

[4] Shinde M.S., Ashtankar K.M. (2017). Additive manufacturing-assisted conformal cooling channels in mold manufacturing processes. Adv. Mech. Eng..

[5] Piekło J., Burbelko A., Garbacz-Klempka A. (2022). Shape-Dependent Strength of Al Si_9_Cu_3_FeZn Die-Cast Alloy in Impact Zone of Conformal Cooling Core. Materials.

[6] Armillotta A., Baraggi R., Fasoli S. (2014). SLM tooling for die casting with conformal cooling channels. Int. J. Adv. Manuf. Technol..

[7] Piekło J., Garbacz-Klempka A. (2020). Use of Maraging Steel 1.2709 for Implementing Parts of Pressure Mold Devices with Conformal Cooling System. Materials.

[8] Piekło J. (2019). Application of SLM Additive Manufacturing Method in Production of Selected Cooling System Elements in Die Casting Molds.

[9] Reggiani B., Todaro I. (2019). Investigation on the design of a novel selective laser melted insert for extrusion dies with conformal cooling channels. Int. J. Adv. Manuf. Technol..

[10] Oskar Frech GmBH Die-Casting Mould Insert with Conformal Cooling.

[11] Mardaras J., Emile P., Santgerma A. (2017). Airbus Approach for F&DT Stress Justification of Additive Manufacturing Parts. Procedia Struct. Integr..

[12] Zhao Z., Dong C., Kong D., Wang L., Ni X., Zhang L., Wu W., Zhu L., Li X. (2021). Influence of pore defects on the mechanical property and corrosion behavior of SLM 18Ni300 maraging steel. Mater. Charact..

[13] Klobčar D., Tušek J. (2008). Thermal stresses in aluminium alloy die casting dies. Comput. Mater. Sci..

[14] Abdulhadi H.A., Aqida S.N., Ishak M., Mohammed G.R. (2016). Thermal Fatigue of Die-Casting Dies: An Overview. MATEC Web Conf..

[15] Piekło J., Maj M., Pysz S. (2013). Experimental-numerical model of the initiation and propagation of cracks in die inserts. Arch. Foundry Eng..

[16] Long A., Thornhill D., Armstrong C., Watson D. (2012). Predicting die life from die temperature for high pressure dies casting aluminium alloy. Appl. Therm. Eng..

[17] Yadolla A., Mahtabi M., Khalili A., Doude H., Newman J. (2018). Fatigue life prediction of additively manufactured material: Effects of surface roughness, defect size, and shape. Fatigue Fract. Eng. Mater. Struct..

[18] Meneghetti G., Rigon D., Gennari C. (2019). An analysis of defects influence on axial fatigue strength of maraging steel. Int. J. Fatigue.

[19] Casati R., Lemke J.N., Tuissi A., Vedani M. (2016). Aging Behaviour and Mechanical Performance of 18-Ni 300 Steel Processed by Selective Laser Melting. Metals.

[20] Tan C., Zhou K., Kuang M., Ma W., Kuang T. (2018). Microstructural characterization and properties of selective laser melted maraging steel with different build directions. Sci. Technol. Adv. Mater..

[21] Jägle E.A., Sheng Z., Kürnsteiner P., Ocylok S., Weisheit A., Raabe D. (2017). Comparison of Maraging Steel Micro- and Nanostructure Produced Conventionally and by Laser Additive Manufacturing. Materials.

[22] EOS GmbH Electro Optical Systems (2022). EOS MaragingSteel MS1, Material Data Sheet.

[23] Concept Laser (2018). CL50WS Maraging Steel 1.2709 (Powder). https://www.ge.com/additive/sites/default/files/2018-12/CLMAT_50WS_DS_EN_US_2_v1.pdf.

[24] Bai Y., Wang D., Yang Y., Wang H. (2019). Effect of heat treatment on the microstructure and mechanical properties of maraging steel by selective laser melting. Mater. Sci. Eng. A.

[25] (2019). Prüfverfahren zur Ermittlung von Fließkurven und Vergleichskennwerten zum Zugversuch Mittels Zerstörungsarmem Prüfeindruck, 3D-Vermessung und Finitelemente Werkstoffmodell.

[26] Walczak J. (1978). Wytrzymałość Materiałów Oraz Podstawy Teorii Sprężystości i Plastyczności Tom II, Strength of Materials and Fundamentals of Elasticity and Plasticity Theory-Volume II.

[27] Piekło J., Garbacz-Klempka A., Burbelko A. (2023). The numerical fatigue life analysis of a conformal HPDC mould core additively manufactured from maraging steel. Materials.

[28] Davies E.A., Connelly F.M. (1959). Stress distribution and plastic deformation in rotating cylinders of strain-hardening material. J. Appl. Mech..

[29] Wierzbicki T., Bao Y., Lee Y.-W., Bai Y. (2005). Calibration and evaluation of seven fracture models. Int. J. Mech. Sci..

[30] Ziółkowski A.G. (2022). Parametrization of Cauchy Stress Tensor Treated as Autonomous Object Using Isotropy Angle and Skewness Angle. Eng. Trans..

[31] Bridgman P.W. (1956). Studies in Large Flow and Fracture.

[32] Peng J., Wang Y., Dai Q., Liu X., Liu L., Zhang Z. (2019). Effect of Stress Triaxiality on Plastic Damage Evolution and Failure Mode for 316L Notched Specimen. Metals.

[33] Bonora N. (1997). On the effect of triaxial state of stress on ductility using nonlinear CDM model. Int. J. Fract..

[34] Kempen K., Yasa E., Thijs L., Kruth J.P., Van Humbeeck J. (2011). Microstructure and mechanical properties of Selective Laser Melted 18Ni-300 steel. Phys. Procedia.

